# Bupropion Increased More than Five Times the Systemic Exposure to Aripiprazole: An In Vivo Study in *Wistar albino* Rats

**DOI:** 10.3390/metabo14110588

**Published:** 2024-10-30

**Authors:** Iulia-Maria Ciocotișan, Dana Maria Muntean, Laurian Vlase

**Affiliations:** Department of Pharmaceutical Technology and Biopharmacy, Faculty of Pharmacy, “Iuliu Hațieganu” University of Medicine and Pharmacy, 41 Victor Babeș Street, 400012 Cluj-Napoca, Romania; iulia.ciocotisan@umfcluj.ro (I.-M.C.); laurian.vlase@umfcluj.ro (L.V.)

**Keywords:** pharmacokinetics, interaction studies, metabolism, aripiprazole, dehydroaripiprazole, bupropion

## Abstract

**Background/Objectives**: In psychiatric disorders, antipsychotics and antidepressant medication are often administered together. Aripiprazole, a third-generation antipsychotic drug, is extensively metabolized by CYP2D6 and CYP3A4 isoenzymes, while bupropion, used in depressive disorders, is known as a moderate or strong CYP2D6 enzyme inhibitor. This in vivo experiment aimed to assess the presence of a pharmacokinetic drug interaction between aripiprazole and bupropion and its magnitude on the systemic exposure of aripiprazole. **Methods**: 24 healthy *Wistar albino* male rats were included in two study groups. A single dose of 8 mg/kg aripiprazole was given to rats in the reference group, while the test group received repeated doses of bupropion for 6 days, followed by a single dose of aripiprazole. An LC-MS/MS method was developed for the concomitant quantification of aripiprazole and its active metabolite, dehydroaripiprazole, and non-compartmental analysis was employed to assess their pharmacokinetic parameters. **Results**: The mean AUC_0-∞_ of aripiprazole increased 5.65-fold (1117.34 ± 931.41 vs. 6311.66 ± 2978.71 hr·ng/mL), the mean C_max_ increased by 96.76% and the apparent systemic clearance decreased over 9-fold after bupropion repeated doses. The exposure to aripiprazole’s active metabolite increased as well, having a 4-fold increase in the mean AUC_0–∞_ (from 461.13 ± 339.82 to 1878.66 ± 1446.91 hr·ng/mL) and a 2-fold increase in the mean C_max_. **Conclusions**: The total exposure to the aripiprazole parent compound and active moiety significantly increased after bupropion pretreatment in this preclinical in vivo experiment. Clinical studies should further establish the significance of this interaction in humans.

## 1. Introduction

Mental disorders like schizophrenia and depression are considered major public health concerns, adding to the global burden of disease. Antipsychotics (APs) represent the central pharmacological option for the treatment of acute episodes and maintenance therapy for schizophrenia and bipolar disorders.

Aripiprazole (ARI) is a frequently used atypical antipsychotic, approved first by the FDA for the treatment of schizophrenia. The oral formulations were later approved for the treatment of bipolar I disorder, adjunctive treatment of major depressive disorder, being the first AP to receive FDA approval for this indication [1], irritability associated with autistic disorder and as a treatment for Tourette’s syndrome [2]. So far, the European Medicines Agency (EMA) only approved its use in schizophrenia and bipolar I disorder [3]. It is used off-label as well for anxiety disorders, attention-deficit hyperactivity disorder (ADHD), psychosis and agitation in dementia or in children and adolescents, substance use disorder, obsessive–compulsive disorder and other personality disorders like borderline personality disorder [4,5,6].

This N-arylpiperazine derivative [7] is considered a dopamine-system stabilizer, being a D_2_/D_3_ dopamine receptor partial agonist. It works as a functional antagonist in areas of overactivity, e.g., it blocks D_2_ receptors in the mesolimbic pathway at high concentrations of dopamine, giving the antipsychotic effect, and as a functional agonist in areas of underactivity. It is also a partial agonist for serotonin 5HT_1A_ and an antagonist for 5HT_2A_ receptors [4,8,9,10]. Due to this novel mechanism of action for an AP, ARI relieves negative, positive and cognitive symptoms in schizophrenia and related disorders, and it is considered a third-generation AP [4,9,10].

ARI has an absolute oral bioavailability (F) of 87%; it binds to plasma proteins >99%; it reaches maximum plasma concentration in 3–5 h and steady-state in 14 days. It goes through minimal pre-systemic biotransformation, but extensive systemic metabolism takes place in the liver via cytochrome P450 (CYP) enzymes [11]. CYP2D6 and CYP3A4 isoenzymes are the major metabolic routes for ARI [12]. CYP3A4 isoenzyme is responsible for N-dealkylation, while both CYP3A4 and CYP2D6 catalyze dehydrogenation and hydroxylation [13,14]. Important to the overall therapeutic effect, the major plasma active metabolite, dehydroaripiprazole (D-ARI), has a similar pharmacologic profile to its parent compound, and together they represent the active moiety (AM) of the drug. At steady-state, D-ARI makes up 40% of the AM area under the concentration–time curve (AUC) in plasma [11]. A metabolite-to-parent ratio of 0.3–0.5 is normally expected [15]. The half-life (t_1/2_) of ARI varies with the type of enzyme metabolizer. E.g., extensive CYP2D6 metabolizers (EMs) have an elimination t_1/2_ of 75 h, while for poor metabolizers (PMs), it reaches 146 h [2]. A time period of 94 h is an average t_1/2_ for the active metabolite [13].

The usual 10–30 mg/day dosing range exhibits a linear relationship with plasma concentration and predicts well dopamine receptor occupancy and the clinical response [9,16]. Variation in ARI plasma levels has a minimal impact on clinical efficacy, but it may predict some adverse drug reactions (ADRs) [9,11]. ARI has a level 2 recommendation for therapeutic drug monitoring (TDM), meaning that patients may benefit from measuring the plasma levels of ARI for problem-solving, like reducing the risk or the presence of ADRs. A therapeutic reference range of 100–350 ng/mL and 150–500 ng/mL for ARI and the total active moiety (AM), respectively, should be considered [15]. A more recent meta-analysis presented the relationship between plasma–brain concentration and clinical results and suggested a therapeutic reference range of 120–270 ng/mL and 180–380 ng/mL for ARI and AM, respectively, to be taken into account for the treatment of schizophrenia and related disorders. Concentrations above the upper limits mentioned above are unlikely to improve treatment response [9], but supra-therapeutic levels may be related to an increased risk of ADRs and intolerance [15].

In patients treated with ARI, the most common side effects like akathisia, extrapyramidal symptoms, nausea, vomiting, constipation, somnolence, sedation, restlessness, insomnia, headache and dizziness were reported [2,17]. Despite having a more favorable metabolic side-effect profile than other APs, due to its novel mechanism of action, weight gain, although minimal, and metabolic disturbances like hyperglycemia are still being reported [2,6,18]. Some of the adverse effects of ARI, like weight gain [6,18], akathisia [1,17], nausea and vomiting [19], extrapyramidal symptoms, somnolence, sedation, tremor and fatigue [2], were found to be dose-related.

Bupropion (BUP), an inhibitor of noradrenaline and dopamine reuptake with aminoketone structure, is approved by the FDA for major depressive disorder and seasonal affective disorder. It is also effective as an aid to smoking cessation treatment and more recently approved, formulated with naltrexone, as an adjunct for weight management [20]. Similar to ARI, it is used off-label in ADHD, including in pediatric patients [5]. BUP has an oral bioavailability of 90%, and although it is transformed mainly by the CYP2B6 enzyme to the major active metabolite hydroxybupropion, together they showed inhibitory effects on the CYP2D6 enzyme, especially in vivo [13,21]. Threohydrobupropion and erythrohydrobupropion are also active metabolites with an important role in CYP2D6 enzyme inhibition; however, more BUP metabolites have been identified and characterized recently [20,21,22].

It is described either as a moderate [23] or strong CYP2D6 inhibitor [20,22] since it increased the area under the plasma concentration–time curve (AUC) of desipramine 5.2-fold [21]. The mechanism of the CYP2D6 inhibition sparked quite some controversy, as the in vitro results showed weak CYP2D6 inhibitory potential for BUP and its metabolites, which could not explain the in vivo outcomes [21]. Sager et al. (2017) remarked that apart from reversible inhibition, BUP and its metabolites downregulate CYP2D6 mRNA expression, which would explain the in vivo magnitude of the interactions with BUP co-medication [20].

Antidepressants (ADs) and antipsychotic agents are frequently used together as an augmentation strategy in different psychiatric disorders [1,24]. Although a series of studies investigating the pharmacokinetic influence of other ADs like paroxetine [25,26], fluoxetine [27,28] and duloxetine [29] on ARI are available, the literature on the co-medication of ARI and BUP from a pharmacokinetic perspective is missing. A retrospective, longitudinal analysis from a large pharmacovigilance study revealed that BUP was one of the most frequently involved drugs in a drug–drug interaction (DDI) among psychiatric patients [30].

The present study aimed to assess the probable metabolic drug–drug interaction between bupropion and aripiprazole from a pharmacokinetic perspective, in vivo, in *Wistar albino* rats.

## 2. Materials and Methods

### 2.1. Materials and Reagents

Dehydroaripiprazole analytical standard (purity 99.66%) was purchased from MedChemExpress (South Brunswick, NJ, USA), haloperidol pharmaceutical primary standard was bought from Merck (Darmstadt, Germany), aripiprazole was used from Astoret^®^ 10 mg orodispersible tablets from Terapia (Cluj-Napoca, Romania) and bupropion hydrochloride was extracted from Elontril^®^ 150 mg extended-release tablets from GlaxoSmithKline plc (Brentford, UK). Heparin sodium 5000 UI/mL from Belmedpreparaty (Minsk, Belarus), ketamine (Vetased^®^ 10% Farmavet, Bucharest, Romania), xylazine (XylazinBio^®^ 2% Bioveta, Ivanovice na Hané, Czech Republic), formic acid 98% and acetonitrile analytical reagent from Merck (Darmstadt, Germany) were purchased.

### 2.2. Dosing and Preparation

Aripiprazole from orodispersible tablets was brought to a suspension in carboxymethyl cellulose 1%. The suspension was vortex-mixed before each administration. We considered 15 mg ARI a usual therapeutic dose in humans. We multiplied by 10 the dose for rats/kg, but a higher dose of 8 mg/kg body weight (b.w.) was chosen to be suitable for detection in rat plasma, considering the analytical method sensitivity. Bupropion solution was obtained by extracting the hydrochloride salt from tablets in distilled water and maintaining its stability at acid pH by adding glacial acetic acid. We administered 43 mg/kg b.w. in rats, multiplying the target dose in humans of 300 mg/day by 10.

### 2.3. Animals

Healthy adult *Wistar albino* male rats, weighing 350 ± 50 g, were obtained from the Experimental Medicine Centre and Practical Skills (Cluj-Napoca, Romania), where the experiment took place. At least a week before study procedures, animals were acclimatized to standard conditions: constant temperature of 21–22 °C, humidity 50 ± 30%, 12 h light–dark cycles, noise reduction, ventilated, cleaned cages. They were given pellet diet and access ad libitum to tap water. The data collected from each rat described a complete pharmacokinetic profile, thus reducing the number of animals used for the experiment.

### 2.4. Ethical Approval

The experiment on animals received approval from the local ethics committee of the Iuliu Hațieganu University and the competent authority in accordance with the specific regulations and amendments of the 43/2014 Law on the protection of animals used for scientific purposes, published in “Monitorul Oficial”, Romania, which transposes Directive 2010/63/EU of the European Parliament and of the Council of 22 September 2010 on the protection of animals used for scientific purposes, published in the Official Journal of the European Union. Ethics committee approval no: 322/02.08.2022.

### 2.5. Experimental Design and Procedures

Twenty-four *Wistar albino* male rats were divided into two groups. Twelve rats received a single dose of ARI 8 mg/kg, representing the reference group. For the test group, each rat (n = 12) was administered daily doses of BUP 43 mg/kg b.w. for 6 days (enough to reach steady-state), followed by administration of a single-dose ARI 30 min after the last dose of BUP on day 6. The rats in both groups were fasted 12 h before ARI single-dose or last dose of BUP, followed by ARI single-dose administration, respectively.

The collection of blood samples was carried out using the BASi Culex ABC^®^-Automatic Blood Collector device (BASi, West Lafayette, IN, USA). This facilitated the collection of exact volumes per sample and at exact times post-administration. One day prior to blood sample collection, rats in both groups went through a surgical procedure consisting of a cannula insertion on the femoral vein under intramuscular anesthesia (0.1 mL/100 g b.w. of 1:1 ketamine and xylazine solution), which allowed the device to have access to the rats’ bloodstream. The excess blood drawn on the catheter was returned to the animal along with saline, replacing the volume of blood collected to avoid fluid depletion.

Blood samples, 200 μL each, were automatically drawn in heparinized Eppendorf tubes at 10, 20, 30, 45 min, 1, 1.5, 2, 2.5, 3, 4, 6, 8, 10, 12, 16, 20, 24 and 30 h after ARI single-dose administration in the two experimental groups, and they were immediately placed at −20 °C until further analysis.

### 2.6. Sample Preparation

Samples were precipitated using acetonitrile. For a 0.1 mL blood sample, 0.3 mL of acetonitrile were added. After the samples were vortex-mixed with IKA Vortex 2 (1000 rpm) for 10 s and centrifuged for 5 min at 10,000 rpm using a Sigma 3-30KS centrifuge (9168× *g*), the supernatant was injected into the HPLC system.

### 2.7. Aripiprazole and Dehydroaripiprazole Assay

For concomitant quantification of ARI and D-ARI, a validated reverse-phase liquid chromatography coupled with tandem mass spectrometry (LC-MS/MS) method was developed. The HPLC system Agilent 1100 series with a binary pump, autosampler and thermostat (Agilent Technologies, Santa Clara, CA, USA) was used. The Bruker Ion Trap SL (Bruker Daltonics GmbH, Bremen, Germany) was used for detection. Chromatographic separation of the analytes was achieved with a Zorbax SB-C18 column (100 × 3.0 mm, 3.5 μm) (Agilent Technologies, Santa Clara, CA, USA). Haloperidol spiked into the thawed blood samples was used as an internal standard. The mobile phase consisted of water and 0.3% (*m*/*v*) formic acid as eluent A and acetonitrile as eluent B, eluted in a linear gradient (start with 20% acetonitrile, up to 2.5 min 38% acetonitrile, keep at 38% acetonitrile until 4.2 min, re-equilibrate with 20% acetonitrile for 2 min). The injection volume was 5 μL, the flow rate was 1 mL/min and the thermostat temperature was set at 40 °C. The MS detection was in multiple reaction monitoring mode (MRM), and the ESI-MS spectra were recorded in positive ion mode. The following mass transitions were used for analyte quantification: *m*/*z* 448 → *m*/*z* 285 for ARI, *m*/*z* 446 → *m*/*z* 285 for D-ARI and *m*/*z* 376 → *m*/*z* 165 for haloperidol. In these chromatographic conditions, the retention times were 2.80 min for haloperidol, 3.15 min for D-ARI and 3.55 min for ARI, respectively. The concentrations of the two analytes were determined by the instrument data system (QuantAnalysis software, Bruker Daltonics GmbH, Bremen, Germany) using peak area ratios versus concentration ratios of each analyte versus internal standard. The calibration curve model was determined for five calibration series by the linear least squares analysis, weighted (1/y2). The intra-day precision, expressed as coefficient of variation, CV %, and accuracy, expressed as the relative difference between obtained and theoretical concentration, bias %, were determined by analysis of five different samples from each QC standard at lower, medium and higher levels on the same day. The inter-day precision and accuracy were determined by analysis on five different days of one sample from each QC standard at lower, medium and higher levels. The lower limit of quantification (LLOQ) was established as the lowest calibration standard with an accuracy and precision of less than 20%. The matrix effects were measured by comparing the response of the spiked plasma with the response of standards in solvent with the same concentration of ARI and D-ARI as in plasma, and for the measurement of the absolute recovery, a spike and a post-spiked sample were compared [31,32]. The calibration curves for both ARI and D-ARI were linear between 2 and 50 ng/mL, with correlation coefficients (r) 0.9942 ± 0.0014 (mean ± S.D., n = 5) for ARI and 0.9981 ± 0.0012 for D-ARI. For ARI, intra- and inter-day precision was less than 9.2%, the accuracy (bias) less than 11.1%, the matrix effects ranged between −8 and 5% and the recovery ranged between 96 and 106%, respectively. For D-ARI, intra- and inter-day precision was less than 11.9%, the accuracy (bias) was less than 13.2%, the matrix effects ranged between −6 and 4% and the recovery ranged between 94 and 103%, respectively.

### 2.8. Pharmacokinetic Analysis

The individual and the mean values of the PK parameters of ARI and D-ARI were obtained by non-compartmental analysis (NCA) using Phoenix Win Nonlin 8.4 software (Pharsight Company, Mountain View, CA, USA). By direct inspection of the data, the maximum plasma concentration (C_max_) and the time to reach the maximum plasma concentration (T_max_) were determined. The individual apparent elimination rate constant (k_el_) was estimated from the log-linear regression from the elimination phase of the analytes’ plasma concentration–time plots. The half-life (t_1/2_) was calculated by dividing 0.693 by the individual k_el_. The area under the curve (AUC) starting from the time of ARI administration to the last determined concentration at 30 h post-administration (AUC_0–30_) was determined using linear trapezoidal integration. The residual AUC, calculated as the last non-zero concentration measured in each animal divided by k_el_, was added to AUC_0–30_ to obtain the total AUC (AUC_0–∞_). Other calculated parameters were the mean residence time (MRT), the apparent volume of distribution (Vz_F) and the apparent total body clearance (Cl_F). The metabolite-to-parent ratio (MPR) was determined individually as the AUC_0–∞_ of D-ARI divided by the AUC_0–∞_ of ARI for both experimental groups and was finally expressed as mean MPR ± S.D.

### 2.9. Statistical Analysis

A one-way ANOVA test was used for statistical analysis to assess the difference between most pharmacokinetic parameters of the two experimental groups after natural logarithmic transformation. For T_max_, Mann–Whitney U, a non-parametric test was applied in SPSS 29.0 software (Chicago, IL, USA). The *p*-values lower than 0.05 were considered statistically significant.

## 3. Results

**Figure 1:**
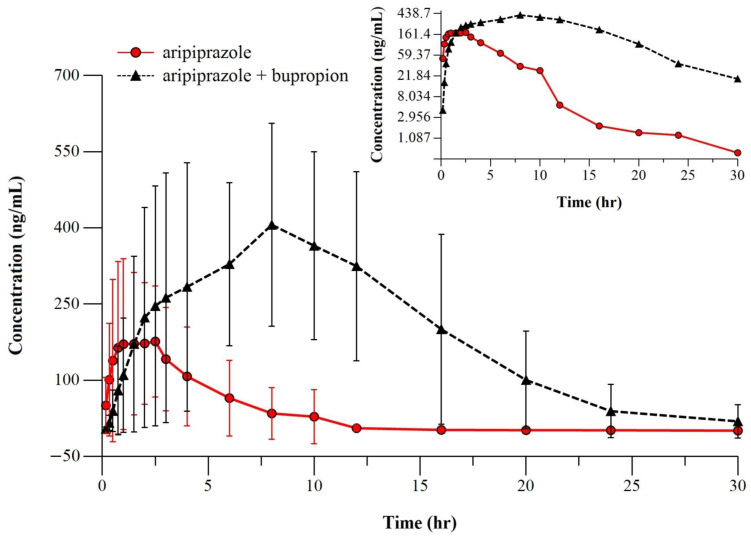
The mean pharmacokinetic profile of ARI after 8 mg/kg single-dose alone (○) (n = 12 rats) or after a previous 6-day treatment with BUP 43 mg/kg (∆) (n = 12 rats). Insert: semi-logarithmic representation.

**Figure 2:**
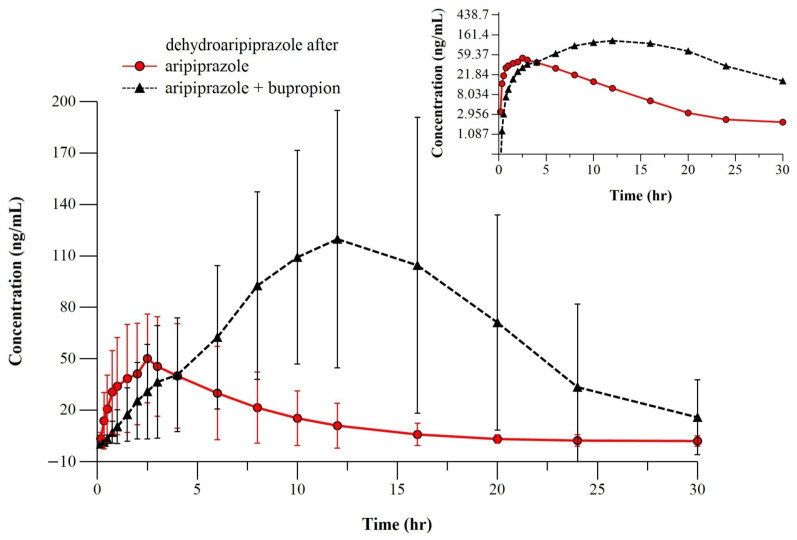
The mean pharmacokinetic profile of D-ARI after ARI 8 mg/kg single-dose alone (○) (n = 12 rats) or after a previous 6-day treatment with BUP 43 mg/kg (∆) (n = 12 rats). Insert: semi-logarithmic representation.

Figure 1 presents the mean plasma profiles of ARI after a six-day pretreatment with BUP 43 mg/kg/day to a single dose of ARI 8 mg/kg (test) and after a single dose of ARI 8 mg/kg alone. Given the same experimental conditions, Figure 2 represents the mean plasma profile of the active metabolite, D-ARI.

The mean ± S.D. values of the pharmacokinetic parameters obtained through NCA for ARI and D-ARI for the two experimental groups are presented in Table 1 and Table 2.

The average value ± S.D. of the individual metabolite-to-parent ratios was 0.43 ± 0.18 for the reference group compared to 0.29 ± 0.11 for the test group, respectively.

## 4. Discussion

The administration of psychopharmacological agents from different therapeutic classes like APs and ADs is a widespread approach to the management of psychiatric disorders. Adjunctive antidepressants may be used to improve the negative and cognitive symptoms of schizophrenia, while APs like aripiprazole are used as adjunctive strategies for antidepressant-refractory depression [1,24,29].

The overall response to antipsychotic therapy may vary depending on the patient’s individual metabolic rate. High inter-individual variability given by the variable expression of the metabolizing enzymes and the risk of DDI recommends ARI for TDM. ARI plasma concentration is highly influenced by CYP2D6 gene polymorphism [9,10], which is why the Dutch Pharmacogenetics Working Group (DPWG) suggested the need for ARI dose adjustment in cases of the CYP2D6 poor-metabolizer (PM) phenotype. In PMs, it is recommended that the dose of ARI should not exceed 10 mg/day for oral administration or no more than 68–75% of the normal or extensive metabolizer’s maximum dose [33]. The other important factor highly influencing the ARI plasma concentration is the concomitant medication [9], like strong CYP3A4 or CYP2D6 inhibitors. When co-medication of ARI and strong CYP2D6 known inhibitors is necessary, half of the usual ARI dose should be administered [2,3].

Dose optimization for ARI is an important concern for clinicians, aiming to increase patients’ safety and adherence to the treatment. Minimizing the ADRs caused by ARI, e.g., those produced by DDIs, is of great interest, knowing that some of them are documented to be dose-related. For instance, increasing doses of ARI are associated with higher rates of akathisia [2]. In a randomized controlled trial (RCT), ARI was most frequently associated with a risk of akathisia and parkinsonism, which has been managed by decreasing the AP dose [17]. Furthermore, a single dose of ARI showed a direct correlation between the AUC_0-t_ of the AP and the rate of nausea and vomiting [19], while a meta-analysis of RCTs found a linear relationship between ARI dose and weight gain. In contrast with most APs, although the weight gain is considered mild for ARI compared to the rest of the APs available, the dose–weight gain curve did not reach a plateau even at higher doses [18]. In addition, a population PK model based on data from children and adolescents found a positive correlation between ARI plasma levels, weight gain and glycated hemoglobin [6].

To our knowledge, the interaction between ARI and BUP has not been investigated so far from a pharmacokinetic perspective, neither in animal models nor in humans, in vivo. We only found two studies that reported some ADRs in patients co-medicated with the two drugs. A case series investigating the efficacy and safety of BUP-resistant major depression augmented with ARI 2.5–10 mg/day reported the presence of akathisia, responsive to dose reduction, and insomnia in some patients [34]. In a post hoc analysis assessing the efficacy, safety and tolerability of long-term treatment with ARI adjunctive to antidepressants, fatigue and somnolence were the most common treatment-emergent adverse effects for BUP + ARI, and fatigue and akathisia for other ADs + ARI. The BUP+ARI combination had a higher mean change in body weight but performed better in terms of fasting glucose and cholesterol compared with other AD + ARI [35].

As for the experiment conducted by us, the administration of a six-day pretreatment with BUP to a single dose of ARI produced significant changes in the PK profiles of ARI, the parent compound, and its active metabolite, D-ARI. The total body exposure increased considerably for ARI, with the mean C_max_ doubling from 239.67 ± 168.59 to 471.59 ± 217.14 ng/mL and the mean AUC_0–30_ and AUC_0–∞_ increasing by 4.85 and 5.65-fold, respectively, after BUP administration. The same tendency of increased exposure was obtained for D-ARI, whose mean C_max_ also increased 2-fold, from 57.68 ± 31.31 to 115.93 ± 59.87 ng/mL, and the mean AUC_0–30_ and AUC_0–∞_ underwent a 4-fold increase each, from 435.81 ± 313.12 to 1739.8 ± 1300.34 hr*ng/mL and from 461.13 ± 339.82 to 1878.66 ± 1446.91 hr*ng/mL.

Taking into account the PK properties of ARI and BUP, the key mechanism involved in this DDI is, most probably, the inhibition of BUP on the CYP2D6 enzyme, highly involved in the biotransformation of ARI at the systemic, hepatic level. Seeing that the exposure of ARI increased by metabolizing-enzyme inhibition, it would be expected to see a decrease in the D-ARI levels due to a reduction in the rate of its formation. However, BUP does not inhibit the other important metabolic pathway of ARI, the CYP3A4 isoenzyme, which is also more important for the transformation of ARI to D-ARI [29]. Thus, we can explain the increase in the C_max_ and AUCs of D-ARI by the increased production from ARI via the CYP3A4-metabolizing pathway.

The CYP3A4 isoenzyme is more important than 2D6 for the formation of D-ARI from the observation that regardless of the CYP2D6 phenotype, the concentrations of the metabolite remain constant [36]. CYP3A4 is the most abundant isoenzyme found in the liver; thus, substantial amounts of D-ARI might be biotransformed from ARI by CYP3A4 when CYP2D6 is less available, like in PM or enzyme-inhibition interactions.

Although there is a twofold difference between the mean values of t_1/2_ for the reference and test groups for ARI, the datasets consisting of the individual values between the two groups are not statistically significantly different. This happened because in the test group some subjects had higher values for t_1/2_, which heavily influenced the mean value of the parameter. Those subjects were not considered outliers because they could have presented polymorphisms of the gene encoding the CYP2D6 enzyme, a poor metabolizer phenotype, explaining the isolated individual high t_1/2_s, which are suggested by the notable S.D. for the mean value of t_1/2_ in the test group.

The differences in the mean and individual values of the t_1/2_ for D-ARI were statistically insignificant when compared to those of ARI. By contrast, D-ARI is not influenced to such an extent by gene polymorphism, the CYP3A4-encoding gene not being highly polymorphic. Since the half-life of D-ARI did not change between the two groups, there are fewer chances that the intrinsic clearance changed for the metabolite. Thus, the decrease in its apparent clearance (Cl_F) is given by an increase in the bioavailability, more specifically, an increased production of D-ARI on the CYP3A4 pathway, due to increased exposure of the parent compound ARI.

ARI has a high oral bioavailability, high plasma protein binding and a low hepatic extraction ratio [14], which means ARI qualifies as a drug with restrictive clearance with BUP pretreatment. As a CYP2D6 enzyme inhibitor, BUP reduced the intrinsic Cl and increased the AUC of ARI. The main mechanism of this PK DDI is expected to take place at the systemic level, in the liver, with CYP2D6 being inhibited by BUP pretreatment, thus reducing the rate of biotransformation of ARI on this metabolic pathway.

The metabolite-to-parent ratio (MPR) obtained for the ARI single-dose group, 0.43 ± 0.18, confirms the information from the current literature that D-ARI represents around 40% of the ARI’s active moiety in the systemic circulation. Considering that the MPR is a measure of the metabolizing enzymes activity, the decrease in the MPR of D-ARI and ARI in the test group versus the reference group should be attributed to CYP2D6 inhibition by BUP, which determined a meaningful increase in the AUC of the parent compound, ARI.

Other metabolizing enzyme-inhibiting antidepressants are known to influence the plasma levels of ARI. In clinical trials on Japanese psychiatric patients, paroxetine significantly increased the plasma concentrations of ARI and its AM by 1.7-fold and 1.5-fold [26]. Escitalopram, a weak inhibitor of CYP2D6, and fluvoxamine, a less potent CYP3A4 inhibitor, did not significantly change the systemic exposure to the AM [26,37].

In a large database for TDM, duloxetine was recently found to increase the plasma concentrations of ARI by 54.2% by inhibiting the biotransformation on CYP2D6 and, probably to a lesser extent, on CYP3A4 pathways [29]. Results from another TDM database showed that ARI combined with fluoxetine or paroxetine resulted in a 45% higher mean dose-adjusted concentration of ARI, with no effect observed on D-ARI. In this case, escitalopram showed slight but statistically significant (*p* < 0.05) changes from the control group for the serum concentrations of ARI and AM, while for mirtazapine, sertraline and venlafaxine co-medication, there was no significant influence [25]. These results are consistent with another routine TDM service, which found that concomitant use of ARI and fluoxetine or citalopram/escitalopram increased the C/D ratio by 44% and 39% compared to the ARI monotherapy group [27].

Lastly, we considered the in vivo *Wistar albino* rat model suitable for the DDI study between ARI and BUP. Rats and humans share cytochrome P450 orthologous proteins, including those for human CYP2D6 and CYP3A4 enzymes, known for their importance in ARI’s metabolism and pharmacokinetic interaction studies; they are CYP2D3 and CYP3A9 in rats, respectively [38]. The in vivo model was chosen as appropriate since the inductive properties of BUP on the CYP2D6 enzyme were not so well expressed in in vitro studies [20,21]. Rodents have a larger liver relative to their body size and body weight (b.w.); therefore, they have a higher proportion of cytochrome P450 enzymes. Rats’ metabolic rate is known to be higher than that of humans [39]. Consequently, we administered higher doses in rats compared to humans, relative to b.w.

Compared to other AP drugs (e.g., clozapine), ARI is considered a drug with a wide therapeutic window. Spina et al. (2016) calculated the therapeutic index for ARI to be 3.3 based on the recommendation on the therapeutic reference range from the Arbeitsgemeinschaft für Neuropsychopharmakologie und Pharmakopsychiatrie (AGNP) group [13,40]. However, patients presenting ARI plasma concentrations higher than the recommended ranges are exposed to a higher risk of ADRs and toxicity [9,15].

The replacement of BUP with other less or non-enzyme-inhibiting AD drugs, as suggested before, to avoid DDI [30], is not always possible. For these cases, measuring the plasma concentration of the victim drug, namely ARI, may be the best strategy to ensure patients’ safety during treatment.

## 5. Conclusions

The administration of BUP significantly increased both ARI and D-ARI total exposure in *Wistar albino* rats. Although animal studies cannot directly be extrapolated to humans, the magnitude of this PK interaction, like the 5.65-fold increase in the mean AUC_0-∞_ and the 2-fold increase in the mean C_max_ of ARI, would justify the continuation of studying the influence of concomitant medication with BUP on the pharmacokinetic parameters and the safety of ARI in clinical trials.

## Figures and Tables

**Table 1:** Pharmacokinetic parameters expressed as mean ± S.D. for aripiprazole (ARI) 8 mg/kg after a single dose alone (reference, n = 12 rats) or in combination with a 6-day bupropion (BUP) 43 mg/kg pretreatment (test, n = 12 rats).

Aripiprazole	Study Group		
PK Parameter (U.M.)	Aripiprazole (n = 12)	Aripiprazole + Bupropion (n = 12)	*p*-Value *
C_max_ (ng/mL)	239.67 ± 168.59	471.59 ± 217.14	0.0049, S
T_max_ (hr)	1.65 ± 0.80	6.92 ± 2.47	<0.0001, S
AUC_0–30_ (hr·ng/mL)	1109.98 ± 931.46	5390.41 ± 2976.52	<0.0001, S
AUC_0–∞_ (hr·ng/mL)	1117.34 ± 931.41	6311.66 ± 2978.71	<0.0001, S
k_el_ (1/hr)	0.15 ± 0.06	0.18 ± 0.10	0.8064, NS
t_1/2_ (hr)	5.56 ± 3.13	11.53 ± 20.25	0.8064, NS
MRT (hr)	4.63 ± 1.78	19.25 ± 27.67	0.0003, S
Cl_F (L/hr/kg)	14.86 ± 15.80	1.57 ± 0.82	<0.0001, S
Vz_F (L/kg)	121.64 ± 143.94	20.67 ± 27.53	0.0004, S

* S—statistically significant when *p* < 0.05; NS—not significant.

**Table 2:** Pharmacokinetic parameters for dehydroaripiprazole (D-ARI) expressed as mean ± S.D. after aripiprazole (ARI) 8 mg/kg single-dose alone (reference, n = 12 rats) or in combination with a 6-day bupropion (BUP) 43 mg/kg pretreatment (test, n = 12 rats).

Dehydroaripiprazole	Study Group		
PK Parameter (U.M.)	Aripiprazole (n = 12)	Aripiprazole + Bupropion (n = 12)	*p*-Value *
C_max_ (ng/mL)	57.68 ± 31.31	115.93 ± 59.87	0.0107, S
T_max_ (hr)	2.25 ± 1.06	10.5 ± 3.21	<0.0001, S
AUC_0–30_ (hr·ng/mL)	435.81 ± 313.12	1739.8 ± 1300.34	0.0006, S
AUC_0–∞_ (hr·ng/mL)	461.13 ± 339.82	1878.66 ± 1446.91	0.0003, S
k_el_ (1/hr)	0.16 ± 0.11	0.14 ± 0.06	0.7584, NS
t_1/2_ (hr)	5.58 ± 2.89	5.79 ± 2.86	0.7584, NS
MRT (hr)	7.61 ± 3.48	14.76 ± 4.31	<0.0001, S
Cl_F (L/hr/kg)	41.64 ± 55.66	7.18 ± 6.22	0.0003, S
Vz_F (L/kg)	238.38 ± 225.81	73.35 ± 115	0.0006, S

* S—statistically significant when *p* < 0.05; NS—not significant.

## Data Availability

All of the data are contained within the article.
